# OA13.03. Promoting safe and integrated maternity care through interprofessional education

**DOI:** 10.1186/1472-6882-12-S1-O51

**Published:** 2012-06-12

**Authors:** A Steel, H Diezel, D Sibbritt, J Adams

**Affiliations:** 1 University of Technology, Sydney and Brisbane, Australia; 2 Embrace Holistic Services, Brisbane, Australia; 3 University of Newcastle, Newcastle, Australia

## Purpose

A majority of women use complementary and alternative medicine (CAM) during pregnancy yet many are not under the care of a qualified CAM practitioner nor inform their primary healthcare clinician of their CAM use. Most conventional maternity carers do not receive training in CAM as part of their formal education and many CAM practitioners do not gain training in order to collaborate effectively with practitioners within the conventional system. Inter-professional education (IPE) is an approach which has been utilised as a tool to promote collaborative practice. However, IPE programs encompassing CAM practitioners and midwives are comparatively scarce. The aim of this study is to evaluate whether participation in an IPE program impacts upon the perceptions and practice behaviours of maternity care providers (midwives and CAM practitioners).

## Methods

Midwives and CAM practitioners participated in discrete workshops designed to promote a shared understanding and develop network, referral and collaboration pathways. The impact of the workshop on the perceptions and practice behaviours of the participants was evaluated using a 3 stage questionnaire: pre-workshop, post-workshop and 3 month follow-up questionnaire.

## Results

Questionnaires were completed by 147 midwives and 30 CAM practitioners. Preliminary analysis identified an improvement in midwives’ perceptions of self-proficiency with regard to CAM particularly with regards to perceived knowledge of CAM (+1.44, p=<0.0001) and ability to answer general questions about safety (+1.45, p=<0.0001) and effectiveness (+1.40, p=<0.0001) of CAM in pregnancy. Final analysis will explore changes in practice behaviours of both midwives and CAM practitioners in the 3 months after completing the IPE workshop

## Conclusion

This study shows IPE may be an effective education tool for midwives and CAM practitioners improving participants’ competence when caring for women choosing to integrate CAM and conventional maternity care during their pregnancy and birth. Further research on the issue of IPE for maternity care providers is suggested.

